# Spatial Patterns and Composition Traits of Soil Microbial Nitrogen-Metabolism Genes in the *Robinia pseudoacacia* Forests at a Regional Scale

**DOI:** 10.3389/fmicb.2022.918134

**Published:** 2022-06-24

**Authors:** Yongli Ku, Yuting Lei, Xiaoting Han, Jieying Peng, Ying Zhu, Zhong Zhao

**Affiliations:** ^1^Key Comprehensive Laboratory of Forestry, Northwest A&F University, Yangling, China; ^2^Key Laboratory of Soil and Water Conservation and Ecological Restoration of State Forestry and Grassland Administration, Shaanxi Academy of Forestry, Xi’an, China; ^3^Key Laboratory of Silviculture on the Loess Plateau State Forestry Administration, Northwest A&F University, Yangling, China

**Keywords:** metagenomics, N-metabolism genes, diversity, *Robinia pseudoacacia*, spatial patterns

## Abstract

Microbial-driven processes related to the nitrogen-metabolism (N-metabolism) in soil are critical for ecosystem functioning and stability. There are spatial patterns of microbial-mediated nitrogen processes, but we still lack an overview of the soil N-metabolism genes of single nitrogen-fixing tree species pure forests at a regional scale. Here, we investigated the spatial variation and drivers of microbial N-metabolism genes in the rhizosphere soil of *Robinia pseudoacacia* on the Loess Plateau by metagenomic technology. We found that the distance-decay of soil N functional gene similarities in *Robinia pseudoacacia* forests on the Loess Plateau spanning a geographic distance of 230 km was significant (*p* < 0.001). The gene composition and co-occurrence patterns in the process of soil microbial N-metabolism were very different, and they were mainly driven by soil pH and MAP (mean annual precipitation). The proportion of positive links and edges co-occurrence networks between N functional genes increased with increasing pH, suggesting that increasing pH promoted connections between functional genes. The relative frequencies of N-metabolism pathways were consistent on the Loess Plateau, the abundance of ammonia assimilation pathway was highest, and the abundance of the nitrogen fixation pathway was the lowest; only the abundance of the nitrogen fixation pathway was not significantly different. The bacterial and archaeal communities involved in soil nitrogen metabolism were significantly different. Structural equation modeling showed that decreases in soil pH and MAP mainly affected the increase in nitrogen functional gene abundance through an increase in the diversity of N-metabolism microorganisms. In conclusion, this study provides a baseline for biogeographic studies of soil microbe functional genes.

## Introduction

Nitrogen is one of the most important nutrient elements and the main limiting factor for plant growth. Nitrogen deficiency reduces photosynthesis, leaf area, green leaf life and plant productivity (Stüeken et al., 2016; Mu and Chen, 2021). Some of the more active nitrogen forms (such as nitrate nitrogen and ammonium nitrogen) can be directly absorbed by plant roots in the soil (Kiba et al., 2011), but this kind of biologically available nitrogen is rare in many environments. The largest freely available nitrogen resource is gaseous nitrogen in the atmosphere (Kuypers et al., 2018), which needs to be transformed for assimilation by plants through the soil microbial nitrogen-metabolism (N-metabolism) network (Moreau et al., 2019). Soil microorganisms play an important role in the N-metabolism of plant. The microorganisms involved in N-metabolism are mainly bacteria, eukaryotes and archaea (Pajares and Bohannan, 2016). They work together to mediate N-metabolism, and the core nitrogen cycle involves six biochemical processes: assimilation, ammoniation, nitrification, denitrification, anaerobic ammonium oxidation and nitrogen fixation (Francis et al., 2007; Harter et al., 2014). With the advancement of research technology, people have conducted more in-depth research on soil N-metabolism in forest ecosystems, from the exploration of microbial processes (Thamdrup, 2012; Philippot et al., 2013) to the study of the related functional genes (Guo et al., 2013; Levy-Booth et al., 2014). At present, metagenomic technology has been effectively applied to the research on functional genes related to N-metabolism (Delmont et al., 2011). This not only allows simultaneous detection of multiple genes involved in the processes of soil nutrient cycling but can also aid in obtaining the genomes of new uncultivated microbial species that are important to soil functions (Bhuiyan et al., 2011).

The spatial pattern of nitrogen functional genes in soil microorganisms and their response factors have been reported. On a global scale, the relative frequencies of nitrogen pathways are consistent among different soil types, and the frequencies of N-metabolism pathways in different soil types are significantly positively correlated (Nelson et al., 2016). However, different environmental factors (such as temperature, etc.) have different effects on microbial N-metabolism genes (Bernal et al., 2012; Niu et al., 2016). Global warming increases the total rate of microbial nitrogen mineralization and nitrification (Zaman and Chang, 2004), but the abundance of the nitrification-related ammonia-oxidizing archaeal *amoA* gene, ammonia-oxidizing bacterial *amoA* gene, and denitrifying *nirK* gene show no significant changes (Dai et al., 2020). Nitrogen fertilization significantly affects soil microbial N-metabolism (Ramirez et al., 2012), and the number of functional genes related to ammoniation, nitrogen fixation, and nitrate assimilation and reduction pathways increase significantly (Chen et al., 2021). The *amoA- b, nirS, narG* and *norB* genes confer the strongest resistance to nitrogen deposition (Luo et al., 2020). However, only few studies have targeted spatial patterns of N functional genes in pure forests with the same tree species at the regional scale.

The Loess Plateau is a typical semiarid area, and one of the most severely eroded areas in the world (Fu et al., 2017). To improve the ecological problems of the Loess Plateau, the Chinese government implemented a large-scale “Grain for Green Project” (GTGP) (Cao et al., 2009). *Robinia pseudoacacia*, with fast-growing and nitrogen-fixing capabilities, is an important afforestation tree species on the Loess Plateau (Liang et al., 2018). And its symbiotic nitrogen fixation rate even reaches as high as 300 kg ha^–1^ year^–1^ in some areas (Liu and Deng, 1991; Cierjacks et al., 2013; Liu et al., 2020). For nitrogen-fixing tree species, when the soil nitrogen level and plant nitrogen resources are low, the root system will recruit rhizobia into the root to form a symbiont (Poole et al., 2018; Concha and Doerner, 2020); when there is sufficient nitrogen in the soil, nodules will degenerate, and plants tend to absorb nitrogen directly from the soil (Goh et al., 2016; Liu et al., 2018a). A study has reported that *Robinia pseudoacacia* on the Loess Plateau lacks the nodules indicating symbiosis at the mature stage (Tatsumi et al., 2020). The same results were obtained in this study, the number of nodules of mature *Robinia pseudoacacia* roots in this area was very small, and the weight of fresh root nodules was only 75 g/m^3^. Therefore, the nitrogen needed for the growth of *Robinia pseudoacacia* at this stage is mainly provided by soil microbial N-metabolism. However, a large number of studies have focused on the occurrence of nodules and the diversity of rhizobia in *Robinia pseudoacacia*, while few reports have been reported on the microbial characteristics of N-metabolism in the rhizosphere soil.

To address these issues, this study explored the characteristics of the microbial community involved in N-metabolism in the rhizosphere soil of mature *Robinia pseudoacacia* on the Loess Plateau by metagenomics. Four counties with large areas of mature *Robinia pseudoacacia* forests were selected for sampling and investigation in the forest distribution area of the Loess Plateau. Our aims were to (1) determine the spatial patterns of soil microbial N-metabolism genes in the rhizosphere of *Robinia pseudoacacia* at a regional scale; (2) identify the dominant factors that regulate microbial N-metabolism genes; and (3) determine composition characteristics of microbial N functional genes and N-metabolizing microorganism in soil at a regional scale.

## Materials and Methods

### Site Description and Sampling

Samples were collected from *Robinia pseudoacacia* forests aged 33–38 years from four counties in the Loess Plateau of China, including Ansai (AS), Baishui (BS), Yongshou (YS) and Zhengning (ZN) (Supplementary Figure 1). The climatic conditions and geographic location information of the sampled areas are shown in Supplementary Table 1. The mean annual precipitation (MAP) and mean annual temperature (MAT) here were the mean MAP and MAT from 1979 to 2020 (from China Meteorological Data Service Center^1^). Six replicate plots of 20 m × 20 m were set up in each county, and three trees were selected for each plot to collect rhizosphere soil samples. The distance between plots was greater than 50 m.

With the tree as the center, within a distance of 1 m, in the four directions of east, south, west and north, the fine roots (diameter < 2 mm) was collected in diameter from 0 to 20 cm of soil using a shovel disinfected with 75% alcohol. The fine roots were then immediately transferred to a sterile sampling bag. The fine roots of three trees were mixed into one sample. The samples were transported to the laboratory on ice. The soil shaken from the fine roots was collected and divided into two parts. One part was used for nitrate nitrogen (NO_3_^–^), ammonium nitrogen (NH_4_^+^) and pH analysis, and the other part was air-dried and passed through a 2 mm sieve for determination of soil properties. After shaking off the soil on the surface of the fine roots, they were placed in a 50 ml sterile centrifuge tube containing sterile water. The test tube was gently shaken, and the soil precipitate was collected by high-speed centrifugation, and stored at −80°C for microbial DNA sequencing analysis. The above indoor operations were carried out under aseptic conditions.

### Soil Chemical Analyses

Soil pH was measured after shaking the soil for 30 min in a water suspension (1:2.5 soil: water ratio). Soil organic carbon (SOC) was analyzed by an Elementar Liqui TOC II analyzer with the removal of inorganic C using hydrochloric acid. The total nitrogen (TN) in the soil was digested by the Kjeldahl method, and then measured with an AA3 continuous flow analyzer. The soil nitrate nitrogen (NO_3_^–^) and ammonium nitrogen (NH_4_^+^) were extracted with 1 mol⋅L^–1^ KCl, and then measured with an AA3 continuous flow analyzer (Liu et al., 2018b). Soil available phosphorus (AP) was extracted with 0.5 mol⋅L^–1^ NaHCO_3_ and then determined by the molybdenum antimony colorimetric method (Manghabati et al., 2018); the total phosphorus (TP) in the soil was determined by the H_2_SO_4_-HClO_4_ digestion method. The total potassium (TK) and available potassium (AK) were measured by the flame photometer method with molten NaOH and NH_4_OAc extraction, respectively (Lcab et al., 2020).

### DNA Extraction and Sequencing

Soil DNA was extracted using the MP Fast DNATM Spin kit (MP Biomedicals, Cleveland, OH, United States) for soil following the manufacturer’s protocol. Prepared DNA samples were used for library preparation and shotgun metagenomics sequencing by Majorbio Company in Shanghai, China^2^. The extracted genomic DNA was detected by 1% agarose gel electrophoresis. The DNA was fragmented using an instrument (Covaris M220), and the fragment length was approximately 400 bp. The NEXTFLEX™ Rapid DNA-Seq Kit (Bio Scientific, United States) was used to construct the paired-end library according to the manufacturer’s instructions. NovaSeq Reagent Kits (Illumina, United States) were used for bridge PCR and sequencing. The specific steps were as follows. (1) One end of the library molecule was ligated with primers and then amplified and immobilized on the chip. (2) The other end of the molecule was randomly complementary to another nearby primer, and was also fixed on the chip to form a ‘bridge’. (3) After PCR amplification, the molecule was linearized into a single strand. (4) We added modified DNA polymerase and 4 kinds of fluorescently labeled dNTPs to synthesize only one base per cycle. (5) The laser scanning reaction plate was used to sequentially read the types of nucleotides polymerized after the reaction of each template sequence to obtain the sequence of the template DNA fragment.

The Illumina sequencing platform can generate billions of reads in a single run, and it is necessary to use statistical methods to control the quality of the measured sequences. Fastp software^3^ was used to control the quality of the raw data. First, the 3′ end and 5′ end of the adapter sequence were cut; second, the reads that were less than 50 bp in length, with an average quality value of less than 20, and contained the N base that represented uncertain base information were removed (Hua et al., 2015). Third, the reads that overlapped with the adapter above a certain portion (length was set up to 15 bp) were removed. In total, approximately 930 million paired reads (read length = 150 bp) were generated, and the number of reads per sample was approximately 51.4 million to 63.2 million (Supplementary Table 2).

The sequences obtained after quality control were short fragment sequences and were assembled using Megahit^4^ (Peng et al., 2012) and Newbler^5^ for multiple hybrid splicing assembly. The open reading frame (ORF) prediction of contigs in the splicing result was performed by using MetaGene^6^ (Hideki et al., 2006). The predicted gene sequences of all samples were clustered using CD-HIT software^7^ (Fu et al., 2012), and the longest gene from each class was taken as the representative sequence to construct a non-redundant gene set. The high-quality reads of each sample were compared with non-redundant gene sets using SOAPaligner software^8^ (Li et al., 2009), and the abundance of genes in the corresponding samples was determined. Diamond software^9^ (Benjamin et al., 2015) was used to compare the non-redundant gene set with the NR database (comparison type: BLASTP). Functional annotation was conducted by aligning sequencing reads against the KEGG database (Release 84.1) (Kanehisa and Goto, 2000) using diamond software (parameter: blastp; E-value ≤ 1e-5).

The related genes of soil microbial N-metabolism were reported in previous literature (Nelson et al., 2015, 2016). From the KEGG database N-metabolism pathway map0091^10^, a total of 60 genes involved in N-metabolism and their corresponding KO numbers were identified, of which 29 were involved in the core nitrogen cycle. The names, functions and classifications of genes related to N-metabolism are shown in Supplementary Table 3. Using Diamond software to compare the gene sequences predicted in the KEGG database with the previously constructed non-redundant gene set, it was found that a total of 49 KO numbers were determined in our samples, and their abundances were assigned. The raw Illumina sequences were deposited in the SRA of NCBI under the BioProject accession number PRJNA799442.

### Statistical Analysis

Non-metric multidimensional scaling (NMDS) (Noval Rivas et al., 2013) analysis and analysis of similarities (ANOSIM) (Clarke, 2010) were performed and graphed by the “vegan” R package after the beta diversity distance matrix was calculated by QIIME software. A simple linear regression model was established in Microsoft Excel to study the relationship between the microbial genetic composition of N-metabolism microbes and environmental factors (i.e., pH and MAP). The variations in rhizosphere soil microbial N-metabolism gene composition and diversity among the four counties were identified by one-way analysis of variance (ANOVA) and least significant difference (LSD) multiple comparison test. Gene co-occurrence networks (Barberan et al., 2012) for microbial N-metabolism genes were structured using the “Igraph” package based on Pearson’s correlation matrix (*p* < 0.05, *r* ≥ 0.7), after which visualization was performed with Gephi-0.9.2. The topological parameters of the resulting networks were also calculated using the “Igraph” package (Lu et al., 2018). The relationships between the abundances of genes and soil properties were tested using Spearman’s rank correlations, and the correlation heatmap was drawn using the “Corrplot” R package. Multiple regression of similarity matrices (MRM) (Mathias et al., 2016; Wang et al., 2017) was used to explore the relationships between the N-metabolism microbial communities and genes and environmental properties using the “Hmisc” and “Vegan” packages. Structural equation modeling (SEM) (Hongwu et al., 2017; Liao et al., 2019) was used to quantitatively analyze the influencing factors of the differences in the microbial gene composition of N-metabolism microbes in the rhizosphere soil of *Robinia pseudoacacia* in different counties. Plspm (Hulland, 1999) is an R package for partial least squares path modeling (PLS-PM) analysis and correlation-based SEM. The latent variable “soil nutrients” was characterized by the three observed variables (TN, NO_3_^–^ and TK) that had the strongest correlation with N-metabolism genes; the latent variable “microbial diversity” was characterized by beta diversity distance; and gene abundance was characterized by the abundance of N-metabolism genes. Both the community histogram and the Venn diagram were calculated and plotted in R v. 4.0.2.

## Results

### Soil Properties

The rhizosphere soil properties of mature *Robinia pseudoacacia* forests are shown in Table 1 in different counties of the Loess Plateau. Except for AP and the N:P ratio, there were significant differences in the other properties. The highest contents of SOC, TN, NH_4_^+^ and AK were observed in ZN County, and the lowest were observed in AS County. The highest TK, NO_3_^–^, C: N ratio and C:P ratio were observed in YS County, and the lowest were in AS County. The lowest pH was observed in AS County, and there were no significant differences among the other three counties.

**TABLE 1:** Soil properties at different sampling sites.

Site name	SOC (g kg^–1^)	TN (g kg^–1^)	TP (g kg^–1^)	TK (g kg^–1^)	NO^3–^ (mg kg^–1^)	NH^4+^ (mg kg^–1^)	AP (mg kg^–1^)	AK (mg kg^–1^)	C:N	C:P	N:P	pH
YS	13.667 ± 2.023b	1.359 ± 0.246a	0.668 ± 0.056ab	14.27 ± 0.943a	30.125 ± 5.326b	13.909 ± 2.632b	2.045 ± 1.534a	0.257 ± 0.038a	10.287 ± 2.055c	20.464 ± 2.483b	2.051 ± 0.429a	8.728 ± 0.052a
ZN	16.626 ± 0.943a	1.288 ± 0.066a	0.696 ± 0.033a	12.433 ± 0.512bc	30.862 ± 2.114b	21.004 ± 4.21a	2.596 ± 0.923a	0.283 ± 0.036a	12.941 ± 1.005b	23.943 ± 2.166ab	1.851 ± 0.108a	8.704 ± 0.054a
BS	16.438 ± 1.339a	1.078 ± 0.128b	0.606 ± 0.104b	13.706 ± 1.782ab	37.312 ± 3.191a	13.054 ± 6.896b	2.208 ± 1.257a	0.18 ± 0.021b	15.321 ± 0.938a	28.147 ± 7.221a	1.845 ± 0.497a	8.552 ± 0.106a
AS	12.667 ± 1.254b	1.034 ± 0.195b	0.611 ± 0.038b	11.204 ± 0.485c	25.756 ± 1.268c	11.322 ± 2.799b	1.319 ± 0.665a	0.196 ± 0.039b	12.626 ± 2.674b	20.77 ± 2.149b	1.713 ± 0.417a	8.280 ± 0.293b

*SOC, soil organic carbon; TN, total nitrogen; TP, total phosphorus; TK, total potassium; NO3^–^, nitrate nitrogen; NH4 + , ammonium nitrogen; AP, available phosphorus; AK, available potassium; C:N. organic carbon to total nitrogen ratio; C:P, organic carbon to total phosphorus ratio; N:P, nitrogen to phosphorus ratio. Values are mean ± SE of six replicates. Within each vertical column, values followed by the same letter are not statistically different, according to Fisher’s protected LSD (P < 0.05).*

### Composition and Relative Abundance of Genes Involved in N-Metabolism

**FIGURE 1:**
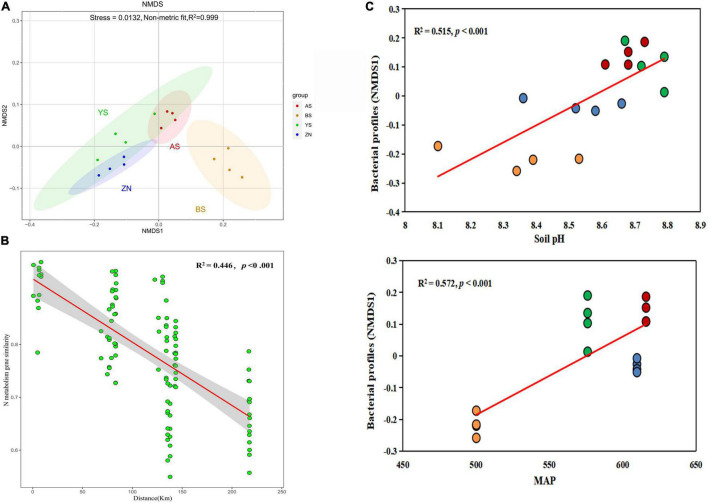
Microbial gene composition for N-metabolism. **(A)** Non-metric multidimensional scaling plots (NMDS) of microbial gene composition for N-metabolism in the rhizosphere soils of *Robinia pseudoacacia* in four counties. **(B)** The distance decay of soil N functional gene similarities based on Bray-Curties distances. **(C)** DistLM indicates that MAP had the closer correlations with gene composition compared with soil pH.

There were significant differences in the microbial gene composition involved in N-metabolism in the four counties on the Loess Plateau (ANOSIM: *p* < 0.05) (Figure 1A). We estimated the distance-decay of soil N functional gene similarities in *Robinia pseudoacacia* forests on the Loess Plateau spanning a geographic distance of 230 km (Figure 1B). The distance decay of soil N functional gene similarities was significant (*p* < 0.001). The variation in gene composition for N-metabolism was more affected by MAP (R^2^ = 0.572) than soil pH (R^2^ = 0.515), as shown by the DistLM analysis (Figure 1C).

**FIGURE 2:**
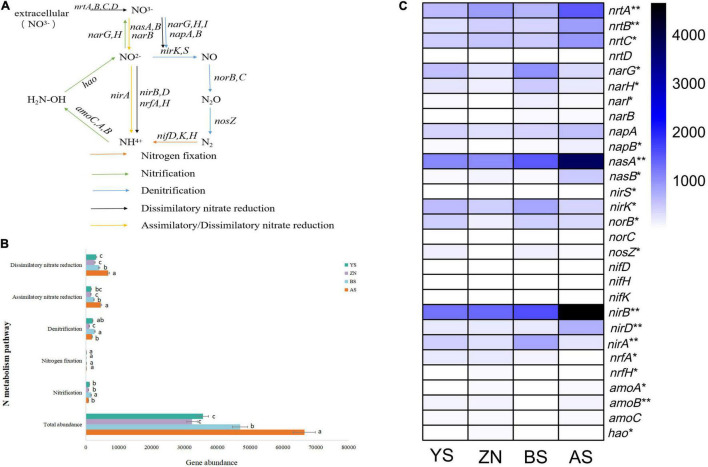
N-metabolism pathways and their frequencies. **(A)** N pathways considered in this study. Arrows of different colors represent different pathways, and the words above the arrows are gene names. **(B)** Abundance of different N-metabolism pathways in *Robinia pseudoacacia* rhizosphere soils in four counties. Different letters on the right of the bars indicate significant differences between treatments, according to Fisher’s protected LSD (*P* < 0.05). **(C)** Abundance of genes related to core N-metabolism pathways. **p < 0.01 and *p < 0.05.

The core nitrogen cycle involves four reduction pathways and two oxidation pathways (see text footnote 10). However, only five of them existed in the rhizosphere soil of mature *Robinia pseudoacacia* forests on the Loess Plateau, namely nitrogen fixation, nitrification, denitrification, dissimilatory nitrate reduction and assimilatory nitrate reduction (Figure 2A). There were significant differences in the total abundance of N-metabolism genes, with AS County having the highest (Figure 2B). Among the five pathways, the abundance of genes in the dissimilatory/assimilatory nitrate reduction pathways were higher, while the nitrogen fixation pathway had the lowest abundance. Except for the nitrogen fixation pathway, there were significant differences in the other pathways among the four counties. There were 29 genes involved in the 5 pathways, among which the genes with high abundance and significant differences were mainly *nirB, nasA* and *nrtA* (Figure 2C). Eighteen other genes involved in N-metabolism were analyzed, among which genes with higher abundance were mainly involved in glutamate metabolism (ammonia assimilation) (Supplementary Table 4).

### Co-occurrence of Microbial Genes Involved in N-Metabolism

**FIGURE 3:**
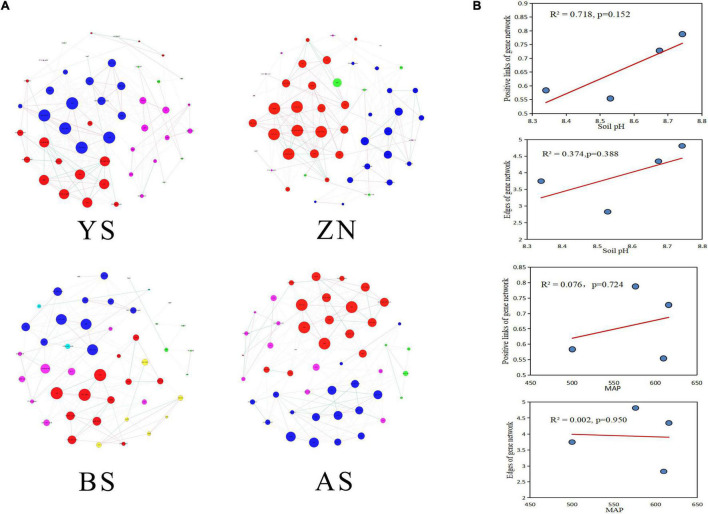
Occurrence networks of genes involved in N-metabolism in rhizosphere soils in four counties. **(A)** The nodes with different colors in the networks represent the dominant modules. **(B)** Scatter plots with a regression curve was used to identify the distribution of the percentage of positive links and the number of edges in the co-occurrence networks of the four counties with the changes of MAP and soil pH.

Co-occurrence networks and the topological parameters of N-metabolism genes showed that the gene co-occurrence patterns were markedly different among the four counties (Figure 3A). The most complex co-occurrence network was for YS County, which had the highest average degree and graph density. The co-occurrence network in AS County was the simplest, with the smallest average degree, graph density, and transfer coefficient (Supplementary Table 5). The positive correlation percentage between N-metabolism gene nodes (R^2^ = 0.718) and the number of edges (R^2^ = 0.374) had a high degree of fit to soil pH with linear regression, generally increasing with increasing soil pH, but the two models did not reach significance. The positive correlation percentage and the number of edges did not fit well with MAP (Figure 3B).

### The Taxonomic Distribution of Genes Involved in N-Metabolism

**FIGURE 4:**
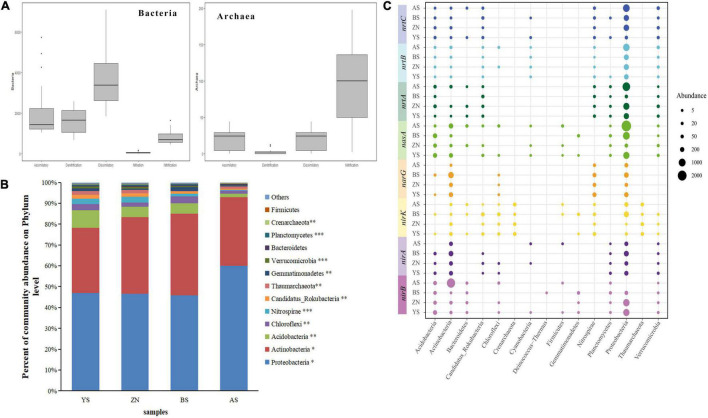
Analysis of nitrogen metabolizing microbial composition in rhizosphere soils. **(A)** Box plot of the frequency of each N pathway for Bacteria and Archaea. The upper and lower bounds of boxes correspond to the 25th and 75th percentiles, with a median line shown. Whiskers represent 1.5*IQ*^R^* (interquartile range). Dots represent outliers. The abscissa axis represents the nitrogen metabolism pathway name. Assimilatory: assimilatory nitrate reduction, Dissimilatory: dissimilatory nitrate reduction, Nifixation: nitrogen fixation. **(B)** Nitrogen metabolizing microbial composition at the phylum level. **(C)** The taxonomic distribution (phylum level) of functional genes. ***p < 0.001, **p < 0.01 and *p < 0.05.

After taxonomic assignment, three domains were identified from N-metabolism functional genes, namely, archaea, bacteria and eukaryotes. The proportions of eukaryotes and archaea were very small, approximately 0.15% and 1.5%, respectively (Supplementary Figure 2). In addition to the nitrogen fixation pathway, bacteria and archaea were both identified in the other four pathways (Figure 4A). Twenty-four phyla were identified, and most phyla had significant differences among the four counties (Figure 4B). The typical genes involved in N-metabolism were annotated to 15 phyla (Figure 4C). All functional genes were found in several phyla; however, they all could be annotated to *Acidobacteria, Actinobacteria* and *Proteobactera*, so these three phyla had a higher number of assigned sequences and were thus considered dominant phyla. Different counties had different distribution ratios of the same gene sequence among the phyla, showing different taxonomic distributions of these functional genes. Among the four pathways of the core nitrogen cycle in the rhizosphere soil of *Robinia pseudoacacia* in the four counties, there were some differences in the number of annotated genera, and the differences in the dissimilation-reduction pathways were relatively small (Supplementary Figure 3A). There were also significant differences in the composition of dominant genera (Supplementary Figure 3B).

There was no significant difference in the Shannon–Wiener index of bacteria in the four counties, but the Chao index was significantly different, with AS County having the highest and ZN County having the lowest value. The Shannon index and Chao index of archaea were significantly different, and the BS County index was lower than those of the other three counties (Supplementary Figure 4A). Microbial community β-diversities were analyzed using NMDS, indicating that the N-metabolism bacterial (stress = 0.029) and archaeal (stress = 0.040) gene composition significantly varied with geographic distance (Supplementary Figure 4B). Since eukaryotes had a low content in the microbial composition and did not participate in the core nitrogen cycle, this group is not discussed again.

### Factors Influencing the Gene Composition of Microbes Involved in N-Metabolism

**FIGURE 5:**
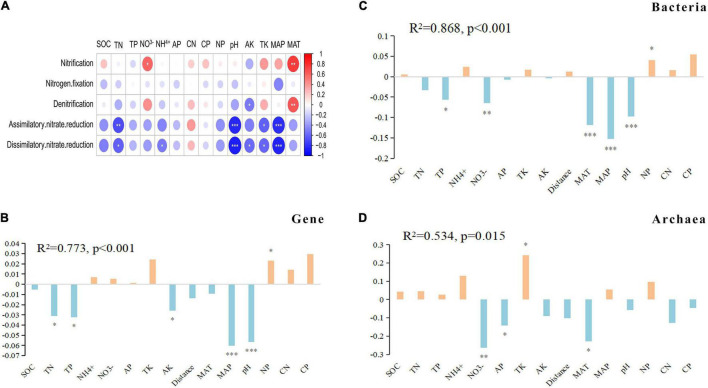
Contributions of environmental properties to the dissimilarities of the genes and microbial communities for N-metabolism. **(A)** The heatmap shows correlations between environmental factors and metabolic pathways. The circle size represents the variable importance. Red and blue represent positive and negative Spearman correlations, respectively. **(B–D)** The bar plots show the coefficient of the multiple regression of dissimilarity matrices (MRM) between the genes and bacterial archaeal communities and environmental properties. ***p < 0.001, **p < 0.01 and *p < 0.05.

The gene abundances of nitrification and denitrification pathways were significantly positively correlated with MAT, dissimilatory/assimilatory nitrate reduction pathway gene abundance was extremely significantly negatively correlated with the MAP and pH, and there was no significant correlation between the nitrogen fixation pathway and soil properties and climatic conditions (Figure 5A).

Correlation analysis of all N-metabolism genes with soil properties and environmental conditions showed that the correlation between each gene and each property index was not the same. In general, there were 30 and 26 genes that had significant correlations with pH and MAP, respectively (Supplementary Figure 5). The MRM method was used to determine the driving factors of N-metabolism genes and microbial community differences. Among the total variation in gene differences, environmental factors, mainly MAP and pH, explained 77.3%. The explanatory rate of environmental factors on the bacterial community (R^2^ = 0.868) was higher than that on the archaeal community (R^2^ = 0.534). The differences in bacterial communities were mainly driven by MAP, pH, MAT and NO^3–^, and the differences in archaeal communities were mainly driven by NO^3–^, AP, TK and MAT (Figures 5B–D).

**FIGURE 6:**
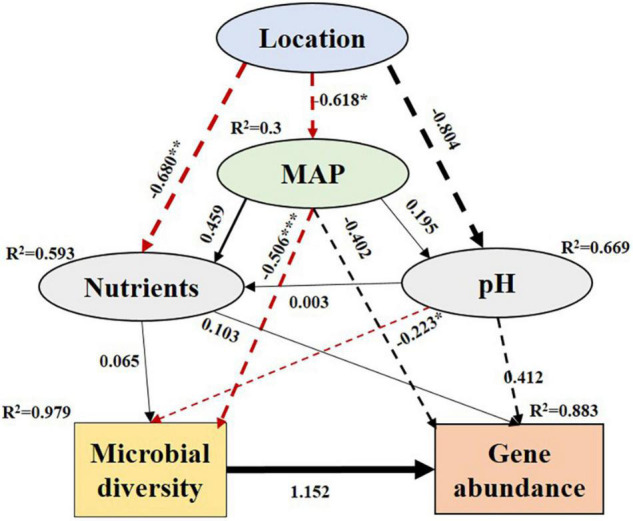
Structural equation model (SEM) illustrating how environmental properties influenced the gene composition for N-metabolism by affecting the diversity of N- metabolism microorganisms. The solid and dashed arrows indicate the positive and negative nature of the path coefficients. The absolute value of the path coefficient corresponds to the line width. Paths that reach a significant level are shown with red lines. Adjacent values near the arrows indicate path coefficients. R^2^ values indicate the proportion of variance explained by each variable. Significance levels are the same as above. ***p < 0.001, **p < 0.01 and *p < 0.05.

Based on the SEM, the four counties in the Loess Plateau had different MAP and soil properties, which in turn affected N-metabolism microorganisms and genes (Figure 6). As the MAP decreased significantly (with an estimated value of −0.618, *p* < 0.05), the diversity of N-metabolizing microorganisms increased significantly (−0.506, *p* < 0.001). At the same time, with the decrease in soil pH, the microbial diversity also increased significantly (−0.223, *p* < 0.05). Microbial diversity had a positive effect on gene abundance (with an estimated value of 1.152). Soil nutrient content also had certain direct and indirect effects on microbial diversity and gene abundance, but the effects were not significant (Figure 6).

## Discussion

Here, we used metagenomic sequencing technology to analyze the characteristics of microbial N-metabolism in the rhizosphere soil of *Robinia pseudoacacia* on the Loess Plateau. The advantage of this method is that it allows us to simultaneously identify traits related to many key functions and organisms with these traits (Myrold et al., 2013). This method also allows us to search for all known genes in one pathway while avoiding primer biases against specific lineages.

The biological process of N-metabolism is a complex interaction of a variety of microorganisms catalyzing different reactions (Marcel et al., 2018). In addition to the core nitrogen cycle, there are other metabolic processes related to core nitrogen cycle intermediate products, such as the metabolism between ammonia and glutamate (Mikeš et al., 1991), ammonia and carbamate, and nitrite and nitroalkane (Héroux et al., 2009). Considering soil N-metabolism overall, the nitrogen fixation pathway in the core nitrogen cycle had the lowest abundance of genes (Figure 2B), and the ammonia assimilation process had the highest abundance of genes (Supplementary Table 4), indicating that the four counties had the same N-metabolism pathway characteristics. This is similar to the results of metagenomic sequencing of microbially mediated N-metabolism pathways worldwide (Nelson et al., 2016). Ammonia assimilation is an ability shared by almost all microorganisms, and sequences in this pathway constitute most N cycle sequences (Nelson et al., 2015). *Robinia pseudoacacia* is a nitrogen-fixing tree species. The nitrogen fixed by root nodule symbionts can enter the soil to participate in the nitrogen cycle (Poblador et al., 2019), so the abundance of nitrogen-fixing pathway genes is low. We did not detect the key genes of the anammox process *hzs* and *hdh*. One possible reason is that the abundance of anammox bacteria may have been too low to be detected, which is supported to some extent by the research report (Ehui et al., 2018; Wang et al., 2021).

Nitrogen-fixing microorganisms were widely distributed in the bacterial and archaeal kingdoms (Figure 4A). Bacteria were present in all pathways, while archaea were only present in specific pathways and were less abundant. The dominant groups in different regions were not very different. Previous studies have shown that the three most abundant phyla of soil nitrogen cycling microorganisms associated with healthy and unhealthy oaks were *Proteobacteria, Acidobacteria* and *Actinobacteria* (Scarlett et al., 2021). In this study, the three predominant phyla of *R. pseudoacacia* rhizosphere soil N-metabolism microorganisms in the four counties were also these three (Figure 4B). More generally, a phylum that is dominant in one habitat is dominant in all habitats. One possible reason for this result is that environmental preferences are preserved below the gate level (Supplementary Figure 4). Our results show that there was a clear environmental preference at the genus level. In the past, research indicators of microbial functions were often the number of community functional groups represented by species, but the disadvantage of dividing functional groups at the species level is that the species belonging to one functional group cannot be divided into another function. Species in the same taxonomic group may have multiple functions at the same time. The results for this study are shown in Figure 4C. Taxonomically distinct microbes coexist with the same functional genes, indicating the existence of functional redundancy (Louca et al., 2018). Environmental change affects not only functional redundancy and community composition, but also the relationship between these organisms (Liu et al., 2021). Since functional redundancy may positively affect community stability and complexity, it provides evidence for the mechanism by which ecosystems respond to changes in microbial communities (Li Y. et al., 2021). Broad environmental adaptations of N-metabolism gene redundancy are critical for N-metabolism in dynamic environments.

The overall structure of microbial nitrogen traits was analyzed, and there were significant differences between the abundances of most genes and pathways involved in the core nitrogen cycle in the four counties (Figure 2 and Supplementary Table 4), showing obvious biogeographical patterns at the regional scale. The taxonomic composition of microorganisms varies spatially (Martiny et al., 2006; Li X. et al., 2021), and this variation can affect ecosystem processes (Strickland et al., 2009; Reed and Martiny, 2013), resulting in spatial variation in functional genes as well. Furthermore, there was a correlation between differences in N metabolism-related microbial community composition and their overall functional differences. Our findings are consistent with similar studies conducted in soil (Fierer et al., 2012a,b). The bacterial and archaeal communities involved in N-metabolism were significantly different (Supplementary Figure 4) and positively correlated with the abundance of N-metabolism genes (Figure 6), and the gene compositions were also significantly different. Niche spaces shared and competed by biome members are fundamental to understanding the coexistence of stable species (Romain et al., 2015). The spatial pattern of nitrogen cycling genes determined the diversity of niches, resulting in significant differences in co-occurrence networks in the four counties.

Environmental filtration, as an ecological selection of abiotic factors, is considered to be a key driver of spatial changes in soil microbial composition and diversity at all scales (Jiao et al., 2020). The taxonomic composition of soil microbes is related to spatial variability in climate, plant diversity, pH, disturbing environmental factors, and many other factors (Liu et al., 2022). The main driving factors of N-metabolism bacterial diversity in the rhizosphere soil of *Robinia pseudoacacia* were MAT, MAP and pH. The main driver of archaeal diversity was the soil nitrate content. These key environmental factors also directly or indirectly affect the composition of functional genes (Tsiknia et al., 2015; Yuan et al., 2018). In the present study, correlation analysis showed that N-metabolism genes and pathways had different correlations with various environmental factors; MRM analysis and linear regression showed that the main factors of differences in overall gene composition were pH and MAP. Once again confirmed that pH reported in previous literature is the main factor affecting various nitrogen cycle genes (Erguder et al., 2010; Prosser and Nicol, 2012; Scarlett et al., 2021). The above-entioned patterns of environmental factors affecting N-metabolism genes directly or indirectly were presented in the structural equations, and MAP and pH significantly affected microbial diversity, which in turn affected gene abundance, but their direct effects on gene abundance did not reach significance.

In summary, this study employed metagenomic techniques, which allowed us to identify all known functional genes of nitrogen cycling, revealing the spatiotemporal pattern of N-metabolism genes in the rhizosphere soil of nitrogen-fixing tree species at a regional scale. There were significant differences in N-metabolism genes and microbial community composition in the rhizosphere soil of *Robinia pseudoacacia* on the Loess Plateau, which were mainly affected by soil pH and MAP. And they mainly affected the abundance of N-metabolism genes by affecting the diversity of N-metabolism microorganisms. The results provide a basis for future trait-based studies on soil nitrogen cycling and a new idea for afforestation and tending of *Robinia pseudoacacia* plantation.

## Data Availability Statement

The datasets presented in this study can be found in online repositories. The names of the repository/repositories and accession number(s) can be found below: https://www.ncbi.nlm.nih.gov/, PRJNA799442.

## Author Contributions

YK: formal analysis, data curation, writing—original draft, and writing—review and editing. YL: formal analysis, writing—original draft, and writing—review and editing. XH: formal analysis and writing—review and editing. JP and YZ: writing—review and editing. ZZ: conceptualization, methodology, supervision, and writing—review and editing. All authors contributed to the article and approved the submitted version.

## Conflict of Interest

The authors declare that the research was conducted in the absence of any commercial or financial relationships that could be construed as a potential conflict of interest.

## Publisher’s Note

All claims expressed in this article are solely those of the authors and do not necessarily represent those of their affiliated organizations, or those of the publisher, the editors and the reviewers. Any product that may be evaluated in this article, or claim that may be made by its manufacturer, is not guaranteed or endorsed by the publisher.

## Footnotes

1
http://data.cma.cn/

2
http://www.majorbio.com

3
https://github.com/OpenGene/fastp

4
https://github.com/voutcn/megahit

5
https://ngs.csr.uky.edu/Newbler

6
http://metagene.cb.k.u-tokyo.ac.jp/

7
http://www.bioinformatics.org/cd-hit/

8
http://soap.genomics.org.cn/

9
http://ab.inf.unituebingen.de/software/diamond/

10
https://www.kegg.jp/entry/map00910
