# Supplementary material for: Spatial Patterns and Composition Traits of Soil Microbial Nitrogen-Metabolism Genes in the Robinia pseudoacacia Forests at a Regional Scale
Source: Front Microbiol. 2022 Jun 24;13:918134. doi: 10.3389/fmicb.2022.918134 (PMC9263705; doi:10.3389/fmicb.2022.918134)
Supplement: Supplementary file 1 [file Data_Sheet_1.DOCX]

**Spatial patterns and composition traits of microbial nitrogen-metabolism genes in the *Robinia pseudoacacia* forests at a regional scale**

**Yongli Ku^1†^, Yuting Lei^1†^, Xiaoting Han^1^, jieying Peng^1^, Ying Zhu^3^, Zhong Zhao^1,2*^**

^1^ Key Comprehensive Laboratory of Forestry, Shaanxi Province, Northwest A&F University, Yangling, 712100, PR China

^2^ Key Laboratory of Silviculture on the Loess Plateau State Forestry Administration, Northwest A&F University, Yangling, 712100, PR China

^3^ Key Laboratory of Soil and Water Conservation and Ecological Restoration of State Forestry and Grassland Administration, Shaanxi Academy of Forestry, Xi'an, 710000, PR China

^†^ These authors have contributed equally to this work and share first authorship

*** Corresponding author:**

Zhong Zhao

E-mail address: zhaozh208@126.com; [zhaozh@nwsuaf.edu.cn](mailto:zhaozh@nwsuaf.edu.cn)


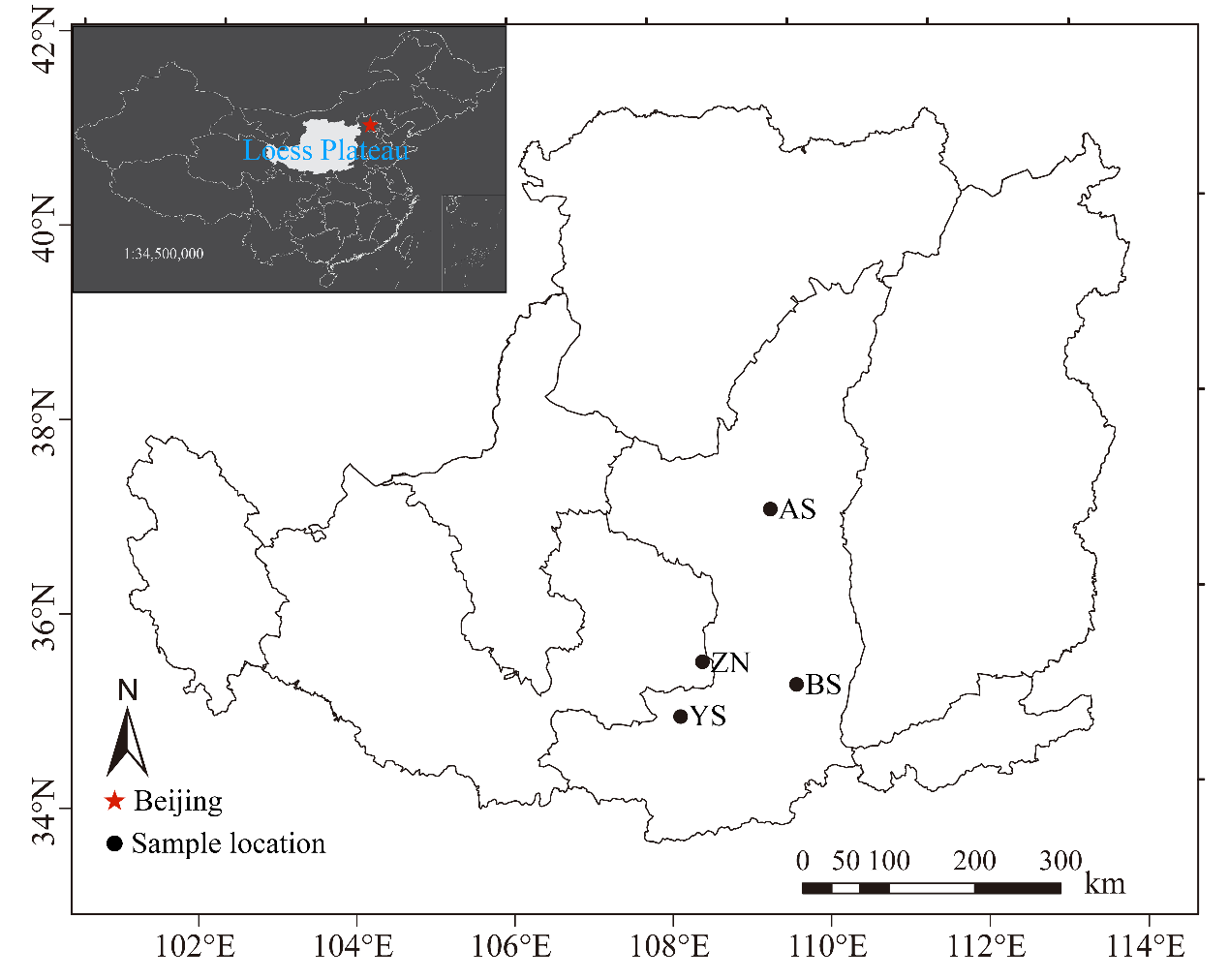


Fig S1 Sampling site locations.


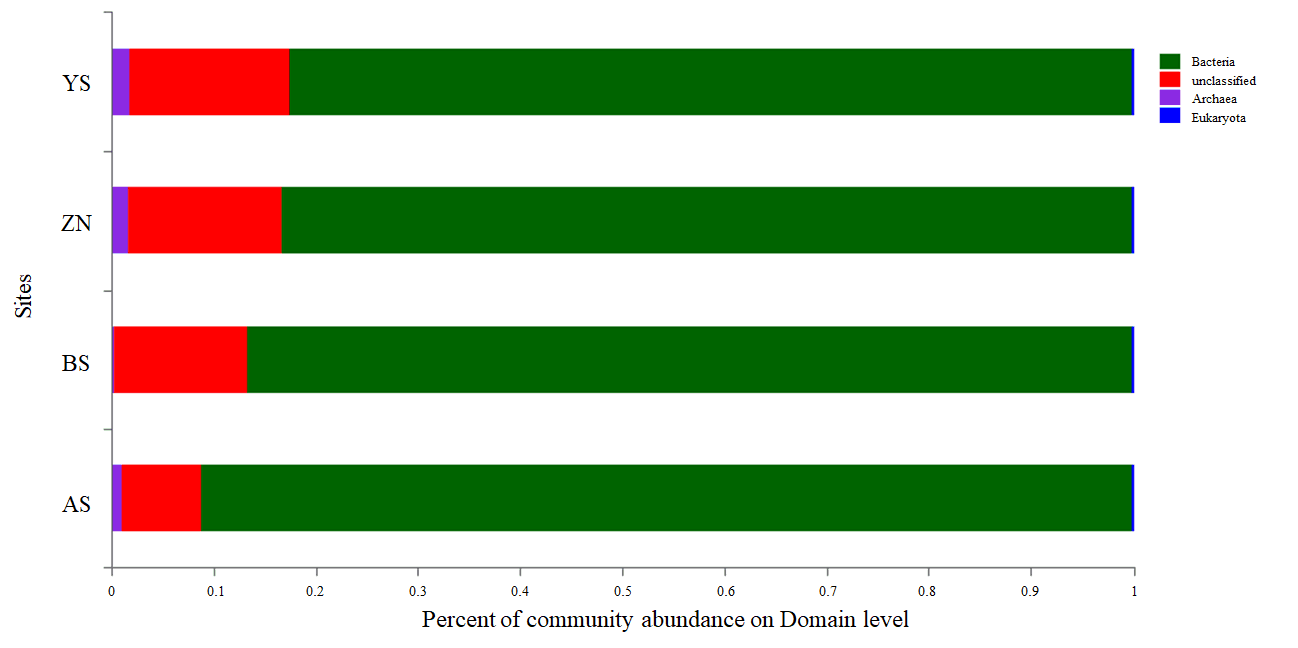


Fig S2 Microbial composition in soil microbial N-metabolism in four counties.


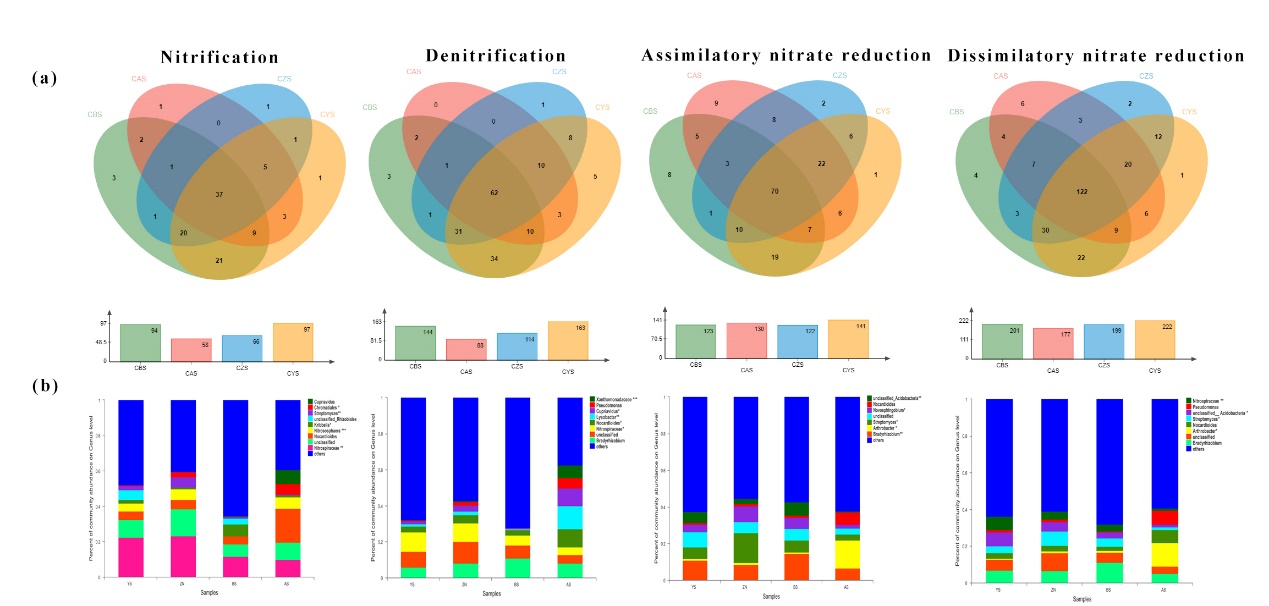


Fig S3 Microbial composition analysis of N-metabolism pathways. (a) Venn diagram counts the number of shared and unique microbial nitrogen metabolism pathways at the genus level in the four counties. (b) Community histogram showing species composition at the genus level in different counties. ***(p<0.001), **(p<0.01) and *(p<0.05)


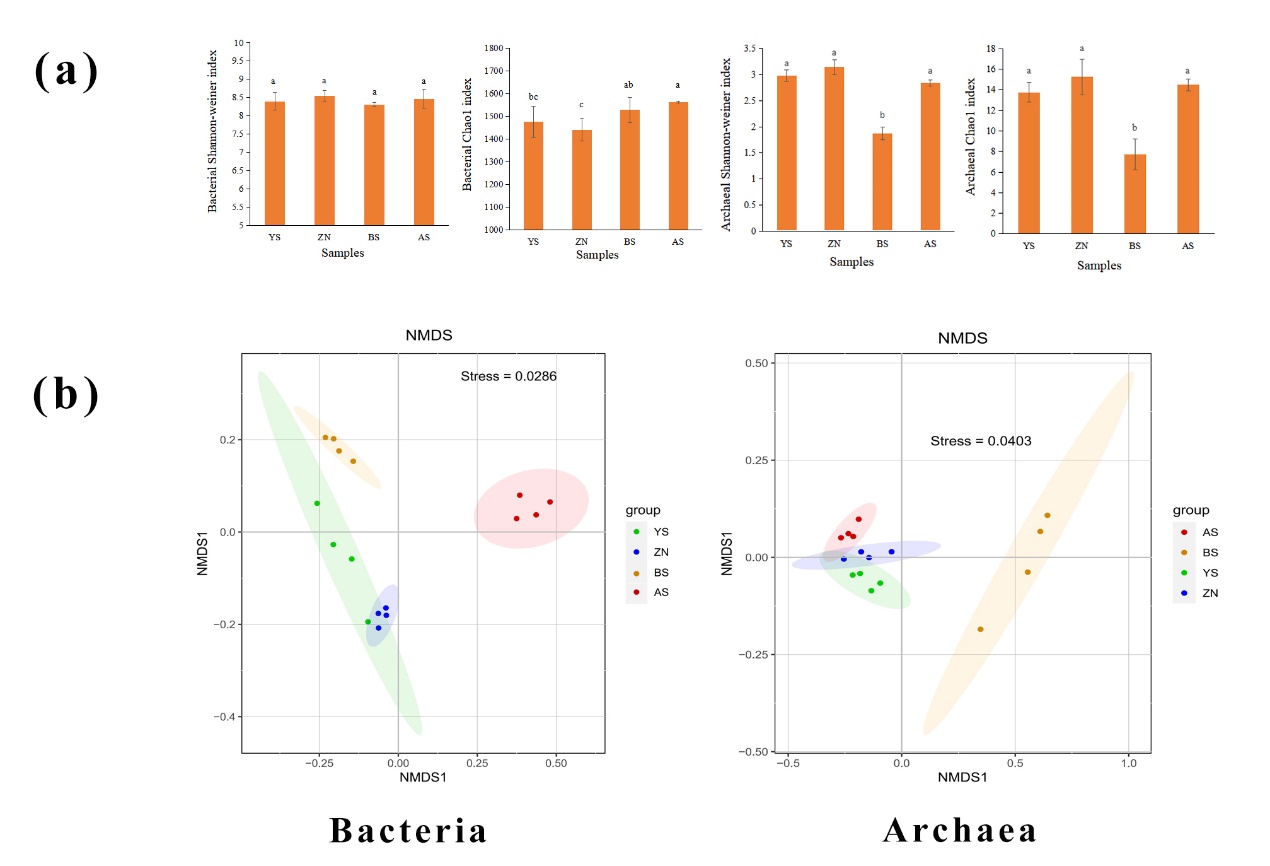


Fig S4 Bacterial and archaeal α-biodiversity (a) and β-biodiversity (b) in the soil

nitrogen cycle. Different letters on the upper side of the bars indicate significant differences between treatments, according to Fisher’s protected LSD (P < 0.05)


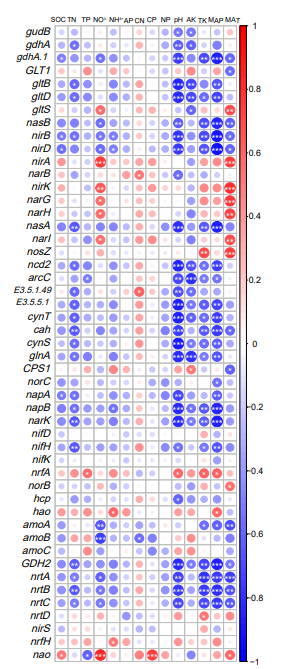
Fig S5 Heatmap of correlations between nitrogen metabolism genes and environmental factors.
