# Supplementary material for: Spatial Patterns and Composition Traits of Soil Microbial Nitrogen-Metabolism Genes in the Robinia pseudoacacia Forests at a Regional Scale
Source: Front Microbiol. 2022 Jun 24;13:918134. doi: 10.3389/fmicb.2022.918134 (PMC9263705; doi:10.3389/fmicb.2022.918134)
Supplement: Supplementary file 2 [file Data_Sheet_2.DOCX]

**Spatial patterns and composition traits of microbial nitrogen-metabolism genes in the *Robinia pseudoacacia* forests at a regional scale**

**Yongli Ku^1†^, Yuting Lei^1†^, Xiaoting Han^1^, jieying Peng^1^, Ying Zhu^3^, Zhong Zhao^1,2*^**

^1^ Key Comprehensive Laboratory of Forestry, Shaanxi Province, Northwest A&F University, Yangling, 712100, PR China

^2^ Key Laboratory of Silviculture on the Loess Plateau State Forestry Administration, Northwest A&F University, Yangling, 712100, PR China

^3^ Key Laboratory of Soil and Water Conservation and Ecological Restoration of State Forestry and Grassland Administration, Shaanxi Academy of Forestry, Xi'an, 710000, PR China

^†^ These authors have contributed equally to this work and share first authorship

*** Corresponding author:**

Zhong Zhao

E-mail address: zhaozh208@126.com; zhaozh@nwsuaf.edu.cn

Table S1 Details of Robinia pseudoacacia forest samples

| Site name | YS | | ZN | BS | AS |
| --- | --- | --- | --- | --- | --- |
| Longitude | | 109°16'-109°45' | 107°56'-108°38' | 107°56'-108°21' | 108°5'-109°26' |
| Latitude | | 35°4'-35°27' | 35°14'-35°36' | 34°29'-34°85' | 36°30'-37°19' |
| MAP (mm) | | 576.089 | 615.938 | 609.746 | 500.182 |
| MAT (℃) | | 12.75 | 10.583 | 13.083 | 10.458 |
| Altitude (m) | | 1150-1200 | 1500-1580 | 1200-1300 | 1250-1360 |
| Soil texture | | Clay loam | Clay | Clay loam | Clay loam |
| Age (Year) | | 38 | 35 | 36 | 33 |
| DBH(cm) | | 23.631±5.932a | 23.56±3.888a | 18.413±4.43c | 17.627±2.63b |
| H(m) | | 16.651±3.776ab | 22.383±27.065a | 14.347±2.719b | 13.127±1.941b |
| CBH(m) | | 10.681±4.209a | 10.73±3.535a | 4.7±0.95b | 4.673±1.476b |
| CD(m) | | 3.989±1.336a | 3.492±0.956a | 3.769±0.743a | 4±0.937a |
| Stand density (stems) | | 21±3.933a | 22±4.147a | 22±4.243a | 22±4.844a |

Stand density: stems/400m^2^; DBH: diameter at breast height; H: height of tree; CBH: clear bole height; CD: crown diameter. Values are mean ± SE of six replicates. Within each vertical column, values followed by the same letter are not statistically different, according to Fisher’s protected LSD (P < 0.05).

Table S2 Summary of sequencing data from four experimental sites.

| Samples | Clean reads | Clean base (bp) | Percent in raw reads (%) | Percent in raw bases (%) |
| --- | --- | --- | --- | --- |
| CZS2 | 61288154 | 9240314285 | 98.05693272 | 97.90650757 |
| CBS4 | 55857032 | 8420999240 | 98.1463792 | 97.99030462 |
| CAS6 | 58088464 | 8758629635 | 98.06509365 | 97.92278791 |
| CBS2 | 55535206 | 8373406297 | 97.89374548 | 97.74887673 |
| CYS2 | 56242320 | 8477214990 | 97.92569411 | 97.74840546 |
| CAS3 | 59779372 | 9009555472 | 98.17184951 | 97.98555141 |
| CZS5 | 52404828 | 7900410851 | 97.89338644 | 97.73604976 |
| CBS1 | 58081920 | 8754674568 | 97.87760904 | 97.70244841 |
| CAS2 | 56354260 | 8495628626 | 97.53388254 | 97.37496925 |
| CBS3 | 57609814 | 8684761154 | 98.25956355 | 98.09780491 |
| CAS1 | 59563588 | 8975856353 | 98.05870603 | 97.85978414 |
| CYS1 | 60485164 | 9113493154 | 98.07180825 | 97.85955685 |
| CYS4 | 61231948 | 9234212406 | 98.02299841 | 97.8977746 |
| CYS5 | 60767542 | 9158062968 | 97.96571613 | 97.77529291 |
| CZS3 | 57013744 | 8594836177 | 98.20870541 | 98.04627099 |
| CZS4 | 63196396 | 9524619322 | 98.14408578 | 97.95858467 |

Table S3 All KEGG Orthology in the N metabolic pathway (KO)

| KO | KO_description |
| --- | --- |
| K00260 | glutamate dehydrogenase [EC:1.4.1.2] |
| K00261 | glutamate dehydrogenase (NAD(P)+) [EC:1.4.1.3] |
| K00262 | glutamate dehydrogenase (NADP+) [EC:1.4.1.4] |
| K00264 | glutamate synthase (NADH) [EC:1.4.1.14] |
| K00265 | glutamate synthase (NADPH) large chain [EC:1.4.1.13] |
| K00266 | glutamate synthase (NADPH) small chain [EC:1.4.1.13] |
| K00284 | glutamate synthase (ferredoxin) [EC:1.4.7.1] |
| K00360 | assimilatory nitrate reductase electron transfer subunit [EC:1.7.99.-] |
| K00362 | nitrite reductase (NADH) large subunit [EC:1.7.1.15] |
| K00363 | nitrite reductase (NADH) small subunit [EC:1.7.1.15] |
| K00366 | ferredoxin-nitrite reductase [EC:1.7.7.1] |
| K00367 | ferredoxin-nitrate reductase [EC:1.7.7.2] |
| K00368 | nitrite reductase (NO-forming) [EC:1.7.2.1] |
| K00370 | nitrate reductase / nitrite oxidoreductase, alpha subunit [EC:1.7.5.1 1.7.99.-] |
| K00371 | nitrate reductase / nitrite oxidoreductase, beta subunit [EC:1.7.5.1 1.7.99.-] |
| K00372 | assimilatory nitrate reductase catalytic subunit [EC:1.7.99.-] |
| K00374 | nitrate reductase gamma subunit [EC:1.7.5.1 1.7.99.-] |
| K00376 | nitrous-oxide reductase [EC:1.7.2.4] |
| K00459 | nitronate monooxygenase [EC:1.13.12.16] |
| K00926 | carbamate kinase [EC:2.7.2.2] |
| K01455 | formamidase [EC:3.5.1.49] |
| K01501 | nitrilase [EC:3.5.5.1] |
| K01673 | carbonic anhydrase [EC:4.2.1.1] |
| K01674 | carbonic anhydrase [EC:4.2.1.1] |
| K01725 | cyanate lyase [EC:4.2.1.104] |
| K01915 | glutamine synthetase [EC:6.3.1.2] |
| K01948 | carbamoyl-phosphate synthase (ammonia) [EC:6.3.4.16] |
| K02305 | nitric oxide reductase subunit C |
| K02567 | nitrate reductase (cytochrome) [EC:1.9.6.1] |
| K02568 | nitrate reductase (cytochrome), electron transfer subunit |
| K02575 | MFS transporter, NNP family, nitrate/nitrite transporter |
| K02586 | nitrogenase molybdenum-iron protein alpha chain [EC:1.18.6.1] |
| K02588 | nitrogenase iron protein NifH |
| K02591 | nitrogenase molybdenum-iron protein beta chain [EC:1.18.6.1] |
| K03385 | nitrite reductase (cytochrome c-552) [EC:1.7.2.2] |
| K04561 | nitric oxide reductase subunit B [EC:1.7.2.5] |
| K05601 | hydroxylamine reductase [EC:1.7.99.1] |
| K10535 | hydroxylamine dehydrogenase [EC:1.7.2.6] |
| K10944 | methane/ammonia monooxygenase subunit A [EC:1.14.18.3 1.14.99.39] |
| K10945 | methane/ammonia monooxygenase subunit B |
| K10946 | methane/ammonia monooxygenase subunit C |
| K15371 | glutamate dehydrogenase [EC:1.4.1.2] |
| K15576 | nitrate/nitrite transport system substrate-binding protein |
| K15577 | nitrate/nitrite transport system permease protein |
| K15578 | nitrate/nitrite transport system ATP-binding protein [EC:3.6.3.-] |
| K15579 | nitrate/nitrite transport system ATP-binding protein |
| K15864 | nitrite reductase (NO-forming) / hydroxylamine reductase [EC:1.7.2.1 1.7.99.1] |
| K15876 | cytochrome c nitrite reductase small subunit |
| K19823 | nitroalkane oxidase [EC:1.7.3.1] |
| K00531 | anfG; nitrogenase delta subunit [EC:1.18.6.1] |
| K01672 | CA; carbonic anhydrase [EC:4.2.1.1] |
| K10534 | NR; nitrate reductase (NAD(P)H) [EC:1.7.1.1 1.7.1.2 1.7.1.3] |
| K15877 | CYP55; fungal nitric oxide reductase [EC:1.7.1.14] |
| K17877 | NIT-6; nitrite reductase (NAD(P)H) [EC:1.7.1.4] |
| K18245 | CA2; carbonic anhydrase 2 [EC:4.2.1.1] |
| K18246 | CA4; carbonic anhydrase 4 [EC:4.2.1.1] |
| K20932 | hydrazine synthase subunit [EC:1.7.2.7] |
| K20933 | hydrazine synthase subunit [EC:1.7.2.7] |
| K20934 | hydrazine synthase subunit [EC:1.7.2.7] |
| K20935 | hdh; hydrazine dehydrogenase [EC:1.7.2.8] |

Table S4 KO abundance table of soil nitrogen cycle at different sampling sites

| KO | KEGG Name | YS | ZN | BS | AS |
| --- | --- | --- | --- | --- | --- |
| K01915 | *glnA, GLUL*** | 8467 | 7500.5 | 11124.5 | 12996 |
| K00265 | *gltB*** | 6486.5 | 5654.5 | 8246 | 9620.5 |
| K15371 | *GDH2 *** | 3661 | 4032 | 5205 | 9851.5 |
| K00459 | *ncd2, npd*** | 1354 | 945.5 | 2244.5 | 3431.5 |
| K00266 | *gltD*** | 1877 | 1766.5 | 2474.5 | 3217 |
| K01673 | *cynT, can** | 1385.5 | 1254 | 2051 | 3210 |
| K00261 | *GLUD1_2, gdhA** | 1642 | 1451 | 1889 | 2000 |
| K00926 | *arcC** | 453.5 | 416 | 671 | 749 |
| K01501 | *E3.5.5.1** | 362.5 | 369 | 446.5 | 629.5 |
| K00284 | *GLU, gltS*** | 598.5 | 398.5 | 892.5 | 549 |
| K01674 | *cah** | 91.5 | 165.5 | 140.5 | 533.5 |
| K01455 | *E3.5.1.49** | 338.5 | 342.5 | 486.5 | 478.5 |
| K01725 | *cynS** | 71 | 78 | 114.5 | 192 |
| K00260 | *gudB, rocG* | 39 | 27 | 57 | 52 |
| K05601 | *hcp* | 3 | 0.5 | 14.5 | 23 |
| K00264 | *GLT1** | 5 | 25 | 1.5 | 5.5 |
| K01948 | *CPS1* | 2.5 | 9 | 0 | 4 |
| K19823 | *NAO* | 1 | 0.5 | 5.5 | 0.5 |

Table S5 Topological metrics of bacterial community networks in soil nitrogen cycle at different sampling sites

| Properties | AS | BS | ZN | YS |
| --- | --- | --- | --- | --- |
| Nodes | 48 | 47 | 49 | 48 |
| Edges | 180 | 133 | 213 | 231 |
| Nodes/Edges | 3.750 | 2.830 | 4.347 | 4.813 |
| Modules | 4 | 6 | 4 | 6 |
| Modularity | 0.531 | 0.580 | 0.489 | 0.426 |
| Diameter | 3.490 | 4.165 | 4.202 | 3.290 |
| Density | 0.160 | 0.123 | 0.181 | 0.204 |
| Transitivity | 0.599 | 0.580 | 0.696 | 0.684 |
| Positive correlation (%) | 41.7 | 46.7 | 27.2 | 21.2 |
| Negative correlation (%) | 58.3 | 53.4 | 72.8 | 78.8 |
